# Altering length and velocity feedback during a neuro-musculoskeletal simulation of normal gait contributes to hemiparetic gait characteristics

**DOI:** 10.1186/1743-0003-11-78

**Published:** 2014-04-30

**Authors:** Karen Jansen, Friedl De Groote, Wouter Aerts, Joris De Schutter, Jacques Duysens, Ilse Jonkers

**Affiliations:** 1 Department of Kinesiology, Human Movement Biomechanics Research Group, KU Leuven, Tervuursevest 101 – box 1501, 3001 Leuven, Belgium; 2 Department of Mechanical Engineering, KU Leuven, Leuven, Belgium; 3 Department of Kinesiology, KU Leuven, Leuven, Belgium; 4 Department of Research, Development & Education, St. Maartenskliniek, Nijmegen, The Netherlands

**Keywords:** Spasticity, Forward simulation, Neuro-musculoskeletal model, Stroke, Gait

## Abstract

**Background:**

Spasticity is an important complication after stroke, especially in the anti-gravity muscles, i.e. lower limb extensors. However the contribution of hyperexcitable muscle spindle reflex loops to gait impairments after stroke is often disputed. In this study a neuro-musculoskeletal model was developed to investigate the contribution of an increased length and velocity feedback and altered reflex modulation patterns to hemiparetic gait deficits.

**Methods:**

A musculoskeletal model was extended with a muscle spindle model providing real-time length and velocity feedback of gastrocnemius, soleus, vasti and rectus femoris during a forward dynamic simulation (neural control model). By using a healthy subject’s base muscle excitations, in combination with increased feedback gains and altered reflex modulation patterns, the effect on kinematics was simulated. A foot-ground contact model was added to account for the interaction effect between the changed kinematics and the ground. The qualitative effect i.e. the directional effect and the specific gait phases where the effect is present, on the joint kinematics was then compared with hemiparetic gait deviations reported in the literature.

**Results:**

Our results show that increased feedback in combination with altered reflex modulation patterns of soleus, vasti and rectus femoris muscle can contribute to excessive ankle plantarflexion/inadequate dorsiflexion, knee hyperextension/inadequate flexion and increased hip extension/inadequate flexion during dedicated gait cycle phases. Increased feedback of gastrocnemius can also contribute to excessive plantarflexion/inadequate dorsiflexion, however in combination with excessive knee and hip flexion. Increased length/velocity feedback can therefore contribute to two types of gait deviations, which are both in accordance with previously reported gait deviations in hemiparetic patients. Furthermore altered modulation patterns, in particular the reduced suppression of the muscle spindle feedback during swing, can contribute largely to an increased plantarflexion and knee extension during the swing phase and consequently to hampered toe clearance.

**Conclusions:**

Our results support the idea that hyperexcitability of length and velocity feedback pathways, especially in combination with altered reflex modulation patterns, can contribute to deviations in hemiparetic gait. Surprisingly, our results showed only subtle temporal differences between length and velocity feedback. Therefore, we cannot attribute the effects seen in kinematics to one specific type of feedback.

## Background

Hemiparetic gait after stroke is characterized by a reduced walking speed and an asymmetric gait pattern, reflected in spatiotemporal parameters, kinematics, kinetics and muscle activations. According to literature, spasticity is a factor often related to these gait deviations [1-4]. However, gait deficits can also result from a range of other impairments, including muscle weakness, motor and sensory dysfunctions, impairments in visuospatial perception and balance problems [2]. The contribution of muscle weakness to an impaired gait pattern has been previously reported in both descriptive and simulation studies. For instance, Mulroy et al. [5] found that maximal isometric torques for dorsiflexors and knee extensors were lowest in post-stroke patients with an ‘extended’ gait pattern, while hip extensor and plantar flexor torques were lower in patients with a ‘flexed’ gait pattern. Hsu et al. [2] showed that gait velocity after stroke was mainly affected by weakness of hip flexors and knee extensors. A simulation study by Jonkers et al. [6] investigated the causal relationship between muscle weakness and resulting deviations in kinematics. From these studies it is clear that muscle weakness influences the post-stroke gait pattern, nevertheless the presence of spasticity in specific muscles will reinforce or counteract specific gait deviations. Spasticity has been clinically defined as “a motor disorder characterized by a velocity-dependent increase in tonic stretch reflexes (muscle tone) with exaggerated tendon jerks, resulting from hyper excitability of the stretch reflex” [7]. Recently, secondary, non-neural changes in muscle parameters are suggested to contribute to a spastic muscle tone as well [8,9]. However, a biomechanical modeling study of Lindberg et al. [10] suggested that the neural component is most dominant in resistance to passive stretch.

The most commonly used clinical measures of spasticity are the (modified) Ashworth or Tardieu scale. However, the validity and reliability of these measures is questionable [11]. Moreover, these scales are not able to differentiate between neural and non-neural factors. Furthermore, they only measure spasticity under passive conditions, while it is known that stretch reflexes are modulated during muscle activation and over different gait phases [9,12-14]. Hence not surprisingly, the spasticity related outcomes of some of these passive tests relate only poorly to characteristics of gait [15].

**Figure 1:**
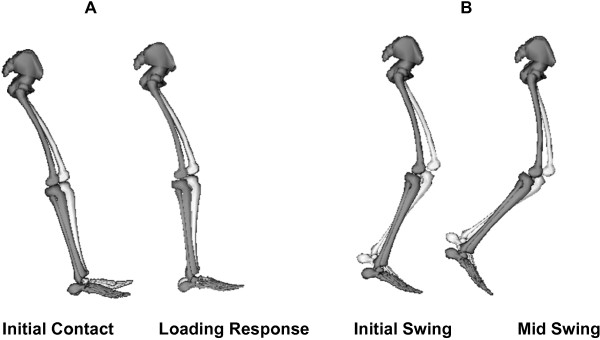
**Kinematic impairments related to spasticity.** The black figure in the front shows the impairments found after stroke (adapted from [18]), compared with a healthy subject (light figure). **A.** Inadequate ankle dorsiflexion and knee hyperextension during initial contact and loading response. **B.** Inadequate knee flexion during the swing phase.

According to Sommerfeld et al. [11] 20-30% of all stroke patients suffer from spasticity. In combination with a worldwide prevalence of stroke of almost 33 million patients [16] this results in at least 6.6 million patients suffering from spasticity. In spite of this large number, the exact contribution of spasticity to gait impairments remains unclear. Spasticity of the rectus femoris (RF) during initial swing has been suggested as the most important mechanism underlying stiff-knee gait, which is characterized by a decreased peak knee flexion during swing (Figure 1B) [4,17,18]. Robertson et al. [4] reported a positive effect of botulinum toxin injection in the RF muscle on stiff knee gait. In addition, quadriceps (QUAD) spasticity is also thought to contribute to knee hyperextension during loading response (Figure 1A) [18]. Plantarflexor (PF) spasticity during the stance phase is suggested to contribute to a lack of dorsiflexion of the foot at first contact and in early stance (Figure 1A). Additionally, the lack of dorsiflexion thrusts the knee into hyperextension during support [1,18]. Studies from Hsu et al. [2] and Lin et al. [3] reported primarily influence of PF spasticity on gait asymmetry. Other studies found no or only weak relations between the clinically measured degree of spasticity and specific movement disorders [9,19,20].

While there is still debate on the involvement of spasticity in hemiparetic gait dysfunction, its pathophysiology is also largely unknown. It is known that gait is generated by a complex interaction of supraspinal (central drive), spinal (central patterns generators) and afferent feedback mechanisms. The afferent feedback mechanisms consist of various type of feedback, originating from muscle spindles (muscle length and velocity feedback), Golgi tendon organs (force feedback) and mechanoreceptors in the joints and in the skin (cutaneous feedback).

It is generally accepted that the muscle spindle afferents are especially involved in spasticity but it is still unclear whether the effects are primarily due to Ia fibers (signaling velocity) or group II afferents (signaling length). Initially, the Ia fibers were thought to be the main responsible fibers [21,22]. However, more recently it was suggested that it is a combined effect of Ia and group II afferents. Nardone and Schieppati [23], Marque et al. [24] and Maupas et al. [25] therefore suggested that a combination of increased excitability of the monosynaptic stretch reflex and polysynaptic length feedback underlies spasticity. Furthermore, it was suggested that a disturbed reflex modulation pattern during gait additionally contributes to the observed gait impairments [26,27].

Models of neurological feedback mechanisms exist with a range of complexity and physiological accuracy, depending on the goal of the study (see reviews [28,29]). Some studies provided very detailed, physiologically realistic models of muscle spindle behavior [30]. However, due to the complexity of these models, they are limited to neural excitation modeling and they do not yet incorporate a musculoskeletal model. Other studies did integrate less complex models of muscle spindles and other types of neural feedback into a dynamic musculoskeletal model to perform simulations [31,32]. However, the main goal of these simulations was to generate a stable gait pattern [31-33] and they only evaluated the contribution of reflex feedback to the overall gait pattern. Paul et al. [32] evaluated the effect of increased reflex gains, but only on the overall gait stability in terms of a stable limit cycle (joint angles versus angular velocity) and not on specific joint kinematics.

In this study a neuro-musculoskeletal model was developed to investigate the contribution of an increased length or velocity feedback and altered reflex modulation patterns to the hemiparetic gait deficits after stroke. To this aim, the classic musculoskeletal model was extended with a neural component that represented muscle spindle feedback pathways for ankle plantarflexors and knee extensors. Due to dynamic coupling, altered feedback and consequent changes in muscle force production of a mono-articular muscle can influence kinematics of joints that the muscle does not span and segments it is not attached to. For bi-articular muscles, the cause-effect relationship between altered feedback, muscle force and the resulting effect on joint kinematics is even more complicated. Therefore, we will differentiate between mono-articular muscles soleus and vasti on the one hand, and gastrocnemius and rectus femoris on the other hand.

To develop the neural model component, H-reflex modulation patterns were recorded or derived from literature to get both base line modulation patterns for the reference subject and altered reflex modulation patterns after stroke Forward simulations using this extended neuro-musculoskeletal model were then generated to investigate the qualitative effect of altered feedback, i.e. the directional effect and the specific phases of the gait cycle where the effect is present.

These results were compared with gait deviations during hemiparetic gait as reported in literature. More specifically, these simulations allow differentiating between the effect of (1) length and velocity feedback, (2) normal and altered reflex modulation patterns and lastly (3) mono- and bi-articular muscles on gait kinematics. We hypothesize that hyperexcitability of the reflex loops can induce gait features characteristic of hemiparetic gait. Furthermore, the presence of altered reflex modulation is hypothesized to further emphasize these features.

## Methods

### Reference simulation

We collected experimental data of a single, healthy subject (age 21 y, mass 54.4 kg) and consequently calculated muscle excitations underlying the experimental kinematics using the workflow described below.

#### *Experimental data*

We registered the three-dimensional trajectories of 34 markers (Krypton, Nikon Metrology NV, Belgium) and ground reaction forces (GRF, see Additional file 1) during walking at 1 km/h on an instrumented treadmill (Forcelink, The Netherlands). The marker protocol consisted of six technical clusters and 16 anatomical markers [34]. Surface EMG data (Zero-wire EMG, Aurion, Italy) were recorded bilaterally at 1000 Hz from 7 muscles: tibialis anterior (TA), lateral gastrocnemius (LGAS), soleus (SOL), lateral vastus (LVAS), rectus femoris (RF), biceps femoris (BF) and semitendinosus (ST). The raw EMG signal was band-pass filtered, root mean square (RMS) values were calculated and the signal was normalized with respect to the maximal amplitude over the gait cycle.

Simultaneously, SOL H-reflexes were recorded during different phases of the gait cycle, to obtain a reference modulation pattern of the muscle spindle feedback. The H-reflex was evoked by a transcutaneous electrical stimulation of the posterior tibial nerve. The applied stimulus was a constant direct current in a rectangular pulse of 250 μsec from a Grass 988 type stimulator. A reference electrode (7 cm by 12.7 cm, self-adhesive, of the Chattanooga group) was placed proximal of the patella. H-reflexes were randomly evoked in one of the 16 phases (bins) of the gait cycle, every three steps and at least ten reflexes were recorded at each bin. To enable a random distribution of the stimuli, an in-house developed Matlab routine was used which allowed for a specific timing of the stimulus with respect to the beginning of the gait cycle (i.e., right heel contact). Heel contact was detected based on the vertical GRF using a threshold of 10% of body weight.

All procedures were approved by the local ethical committee, and the subject gave his informed consent prior to data collection.

#### *Calculate muscle excitations*

Muscle excitations underlying the experimentally measured kinematics were calculated using a standard workflow in OpenSim [35]. In a first step a generic musculoskeletal model (27 degrees of freedom) [36] was scaled to fit the subjects’ anthropometry. An in-house developed Kalman smoothing algorithm implemented in the OpenSim framework used the complete marker trajectories to calculate joint kinematics (see Additional file 2) during walking [37]. A residual reduction algorithm resolved dynamic inconsistencies between the model kinematics and the measured GRF [38]. Computed muscle control (CMC) [38] was then used to compute the muscle excitations $\left(u_{\mathrm{CMC},m}^{\mathrm{ref}}\right)$ that track the experimental kinematics of the healthy subject. After amplitude normalization, the patterns of the calculated muscle excitations were qualitatively compared to the measured EMG to verify the validity of the simulations (Additional file 3).

### Definition of neural control and foot-ground contact model

**Figure 2:**
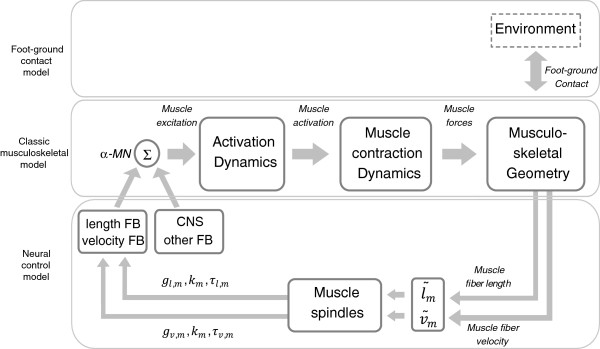
**Schematic diagram of the neuro-musculoskeletal model.** Parameters *g*_*l,m*_ and *g*_*v,m*_ are the gains of the length and velocity feedback of muscle *m,* τ_*l,m*_ and τ_*v,m*_ are the time constants and *k*_*m*_ is the reflex modulation factor of muscle *m* , which is a function of the gait cycle. ${\overset{˜}{l}}_{m}$ and ${\overset{˜}{v}}_{m}$ are the normalized muscle length and velocity. The α-motor neuron (α-MN) receives input from the muscle spindle feedback pathways (length and velocity FB), the central nervous system (CNS) and other non-modeled feedback pathways (other FB).

The effect of an increased feedback and modified reflex modulations on kinematics was investigated using forward simulations. Therefore, the classic musculoskeletal model was extended by a neural control model and a foot-ground contact model (Figure 2).

#### *Neural control model*

In our model, we focused on the contribution of muscle length and velocity feedback to gait kinematics. Therefore we only implemented a simplified model of the muscle spindle feedback pathways. We investigated the effect of reflex hyperexcitability of four muscle groups: (1) SOL, (2) medial and lateral GAS, (3) medial, intermediate and lateral vasti (VAS) and (4) rectus femoris (RF).

The neural excitation *y*_*m*_ of the muscle *m* was therefore modeled as follows:

(1) $$u_{\mathrm{tot},m}=u_{b,m}+u_{l,m}+u_{v,m}$$

(2) $$y_{m}=\;\left\{\begin{array}{c} u_{\mathrm{tot},m}\;0\;\leq u_{\mathrm{tot},m}\;\leq \;1\\ \;1\;u_{\mathrm{tot},m}>1 \end{array}\right)$$

**Figure 3:**
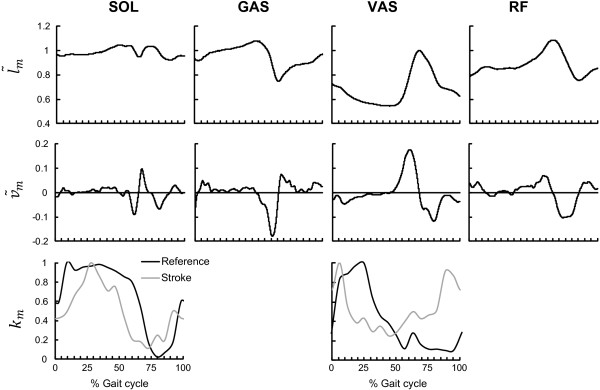
**Normalized fiber length/velocity curves and reflex modulation factors over the gait cycle.** Normalized muscle fiber length (${\overset{˜}{l}}_{m}$, top pane) and muscle fiber velocity (${\overset{˜}{v}}_{m}$, middle pane) for the reference condition are shown as function of the gait cycle for soleus (SOL), gastrocnemius (GAS), vastus (VAS) and rectus femoris (RF). The left lower pane shows the plantarflexors reflex modulation factor (*k*_*m*_) for reference (black, experimentally measured) and stroke subjects (grey, according to Yang et al. 1991). The right lower pane shows the modulation curves for the quadriceps muscle, for reference (black) and stroke (grey) subjects, both according to Faist et al., 1999. The most prominent difference between stroke and control was the lack of suppression of plantarflexors H-reflexes and of quadriceps tendon jerk reflex in the swing phase. Intervals indicated on the X-axis correspond to specific phases of the gait cycle: 0-5%: Initial Contact, 5-20% Loading Response, 20-35%: Mid Stance, 35-50%: Terminal Stance, 50-70%: Pre-Swing, 70-80%: Initial Swing, 80-95%: Mid Swing, 95-100%: Terminal Swing.

Where *u*_*l,m*_ and *u*_*v,m*_ are the length and velocity feedback signals from muscle *m* and *u*_*b,m*_ is the muscle base excitation originating from the central nervous system and the non-modeled feedback pathways. The length feedback *u*_*l,m*_ is modeled by:

(3) $$\;\tau_{l,m}{\overset{˙}{u}}_{l,m}=\left\{\begin{array}{l} -u_{l,m}+k_{m}g_{l,m}{\overset{˜}{l}}_{m}\;{\overset{˜}{v}}_{m}>0\\ -u_{l,m}\;{\overset{˜}{v}}_{m}\;\leq \;0 \end{array}\right)$$

where *g*_*l,m*_ is the gain of the length feedback of muscle m, τ l,m is the time constant and ${\overset{˜}{l}}_{m}$ and ${\overset{˜}{v}}_{m}$ are the normalized muscle length and velocity (Figure 3, top and middle pane). The velocity feedback uv,m is modeled by:

(4) $$\tau_{v,m}{\overset{˙}{u}}_{v,m}=-u_{v,m}+k_{m}g_{v,m}\max\left(0,{\overset{˜}{v}}_{m}\right)$$

where *g*_*v,m*_ is the gain of the velocity feedback of muscle *m* and τ_*v,m*_ is the time constant. There is only length and velocity feedback when the muscle is lengthening. Both *g*_*l,m*_ and *g*_*v,m*_ are modulated by a reflex modulation factor *k*_*m*_ (Figure 3, bottom pane) that is function of the gait cycle.

### Parameter definition of the neural control model for use in the reference simulation

In the reference simulation, part of the previously calculated muscle excitations $u_{\mathrm{CMC},m}^{\mathrm{ref}}$ (see 2.1.2.) was assumed to originate from the central nervous system and non-modeled feedback pathways $\left(u_{b,m}^{\mathrm{ref}}\right)$, while the other part results from length and velocity feedback excitations $\left(u_{l,m}^{\mathrm{ref}}+u_{v,m}^{\mathrm{ref}}\right)$. Reference length and velocity gain factors ( *g*_*l,m*_ and *g*_*v,m*_ ) were determined to generate length and velocity feedback excitations, while constraining the total sum of the base signal and the feedback signals $\left(u_{\mathrm{tot},m}^{\mathrm{ref}}\right)$ to equal the calculated muscle excitations $\left(u_{\mathrm{CMC},m}^{\mathrm{ref}}\right)$.

(5cf.1) $$u_{\mathrm{CMC},m}^{\mathrm{ref}}=u_{\mathrm{tot},m}^{\mathrm{ref}}=u_{b,m}^{\mathrm{ref}}+u_{l,m}^{\mathrm{ref}}+u_{v,m}^{\mathrm{ref}}$$

The reflex modulation factor *k*_*m*_ for SOL and GAS is based on the experimental H-reflex measurements. Peak-to-peak amplitudes of the H-reflex were calculated and reflexes occurring in the same bin were averaged. Data were then normalized to obtain values between 0 and 1, and a spline was used to convert the discrete values into a continuous function. The reflex modulation of the quadriceps is based on tendon jerk reflex measurements reported by Faist et al. [26]. Time constants (τ_*l,m*_,τ_*v,m*_) were based on values from literature (PF, see Reference [39], QUAD, see Reference [40]) (Table 1).

**Table 1:** Parameters neural control model

**Muscle group**	**τ**_***l,m***_	**τ**_***v,m***_	***g***_***l,m ***_**(reference)**	***g***_***v,m***_**(reference)**	***g***_***l,m***_**(stroke)**	***g***_***v,m***_**(stroke)**
Soleus	75 ms	40 ms	0.1	0.59	0.3	1.77
Gastrocnemius	75 ms	40 ms	0.1	0.77	0.3	2.30
Vasti	50 ms	40 ms	0.1	0.37	0.3	1.11
Rectus Femoris	50 ms	40 ms	0.1	0.82	0.3	2.45

### Parameter definition of the neural control model for use in the hemiparetic simulations

Signals from the central nervous system and non-modeled feedback pathways in stroke patients $\left(u_{b,m}^{\mathrm{stroke}}\right)$ were considered to be equivalent with the reference simulation $\left(u_{b,m}^{\mathrm{ref}}\right)$ and were calculated using formula (5):

(6) $$u_{b,m}^{\mathrm{stroke}}=u_{b,m}^{\mathrm{ref}}=u_{\mathrm{CMC},m}^{\mathrm{ref}}-\;u_{l,m}^{\mathrm{ref}}-\;u_{v,m}^{\mathrm{ref}}$$

Spasticity in stroke patients was simulated by multiplying gain factors *g*_*v,m*_ and/or *g*_*l,m*_ with respectively 1.1, 1.2, 1.5, 3 and 6, resulting in an altered length $\left(u_{l,m}^{\mathrm{stroke}}\right)$ and velocity $\left(u_{v,m}^{\mathrm{stroke}}\right)$ feedback signal. However, as all increased feedback gains resulted in a similar directional effect on the kinematics we only report the effects of one increased gain factor i.e. factor 6 (Table 1). Similarly, the effects of the combination of increased length and velocity feedback were not explicitly reported, as the qualitative effect of both remained the same.

(7) $$u_{\mathrm{tot},m}^{\mathrm{stroke}}=u_{b,m}^{\mathrm{ref}}+u_{l,m}^{\mathrm{stroke}}+u_{v,m}^{\mathrm{stroke}}$$

The reflex modulation factor *k*_*m*_ of the quadriceps is based on tendon jerk reflex measurements in hemiparetic spastic patients reported by Faist et al. [26]. The reflex modulation factor *k*_*m*_ for the PF is based on H-reflex modulation patterns in spastic paretic subjects as reported by Yang et al. [27]. Neural control parameters are listed in Table 1.

#### *Foot-ground contact model*

To account for the differences in interaction between the foot and the ground, due to the changes in kinematics when feedback gains are altered, the foot-ground contact was described by an elastic foundation contact model [41]. The contact geometry is described by three spheres attached to the calcaneus segment of both feet (one at the heel and two at the level of the metatarsal arch) and by a contact plane which was attached to the treadmill surface. In an optimization procedure the locations of the spheres (X-Y-Z coordinates) in the calcaneus’ reference frames were optimized by minimizing the kinematic tracking error during a forward simulation using muscle excitations calculated by CMC. Foot-ground contact parameters are listed in Table 2.

**Table 2:** Sphere locations and contact parameters of foot-ground contact model

	**Sphere number**	**Sphere locations (mm)**			**Sphere radius (mm)**	**Stiffness (MPa/m)**	**Dissipation (s/m)**	**Static friction**	**Dynamic friction**	**Viscous friction (s/m)**
		**X**	**Y**	**Z**						
**R**	1	18	11.9	8.2	30	1.86	1000	0.101	0.2	0.01
	2	171	1.4	24.9	15	1.86	1000	0.101	0.2	0.01
	3	139	1.5	38.6	15	1.86	1000	0.101	0.2	0.01
**L**	4	6	3	3.9	30	1.86	1000	0.101	0.2	0.01
	5	184.5	0.8	23.8	15	1.86	1000	0.101	0.2	0.01
	6	142.6	4.5	40.0	15	1.86	1000	0.101	0.2	0.01

R: right, L: left.

### Forward simulations

**Figure 4:**
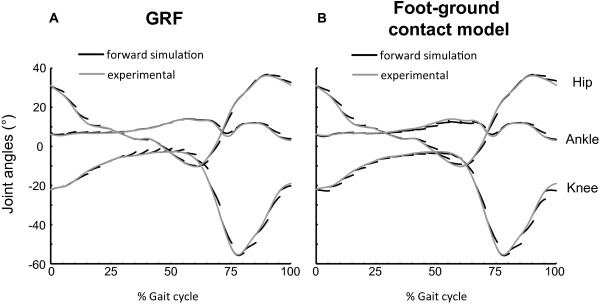
**Comparison of forward simulations using ground reaction forces or the foot-ground contact model. A.** Joint kinematics generated by forward simulations using measured ground reaction forces (GRF) (black) compared with the experimentally measured joint kinematics (grey) as function of the gait cycle. **B.** Joint kinematics generated by forward simulations using the foot-ground contact model (black) compared with the experimentally measured joint kinematics (grey) as function of the gait cycle. Only minor differences in joint kinematics were found between forward simulations using the ground reaction forces and the foot-ground contact model.

Reference kinematic data were then generated through a forward simulation using the extended neuro-musculoskeletal model with the reference gains and reflex modulation patterns as parameters and the base reference excitations (*u*_*b*,*m*_(*ref*)) as input. The experimentally measured GRF were replaced by the force calculated using the foot-ground contact model during the forward simulation (Figure 2). We generated forward simulations for intervals consisting of 5% (~0.1 s) of the gait cycle. This time interval was chosen to limit integration errors arising from round-off and truncation during the open-loop forward simulation. However this interval still allows kinematic changes induced by increased feedback or altered modulation patterns. To evaluate the validity, the reference simulation was compared with the forward simulations over the same time intervals using the measured GRF and with the experimentally measured joint kinematics (Figure 4). Only minor differences were found between the two types of forward simulations and the experimentally measured kinematics.

To evaluate the effect of altered feedback on joint kinematics and on total muscle excitations, the forward simulation was repeated with the same base excitations, but with increased gain factors and altered reflex modulation patterns.

The initial kinematics for each interval were the reference kinematics of the control subject at the corresponding time instant. The initial length and velocity feedback excitation for each interval was the feedback excitation at the end of the previous time interval as this cannot be determined instantaneously. To compensate for the discontinuity this induces between the kinematics and the feedback excitations, we started the simulations slightly before the intended start time of the simulation (i.e. the time instance starting from which we want to present the kinematics of the simulation) and use an overlap (1%) between the successive simulation intervals. This way, the feedback signals will have evolved to the kinematics of the simulation interval of interest.

The mean differences between the simulated reference and stroke joint angles and muscle activations were calculated for each of the ten intervals.

## Results

**Figure 5:**
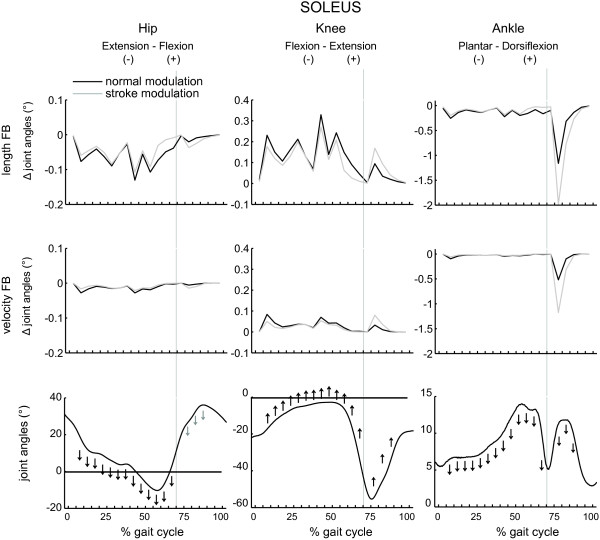
**The effect of increased feedback and altered modulation patterns of soleus on joint kinematics.** The mean differences are shown between the reference and stroke kinematics for increased length and velocity feedback (FB), with normal modulation patterns (‘normal’, black line) or with altered modulation patterns (‘stroke’, grey line), as function of the gait cycle. The bottom panes show the reference kinematics (calculated with CMC), the black arrows indicate the direction of changes for increased length/velocity feedback. Grey arrows show the additional effects of the altered modulation patterns. The grey vertical line indicates stance-to-swing transition.

**Figure 6:**
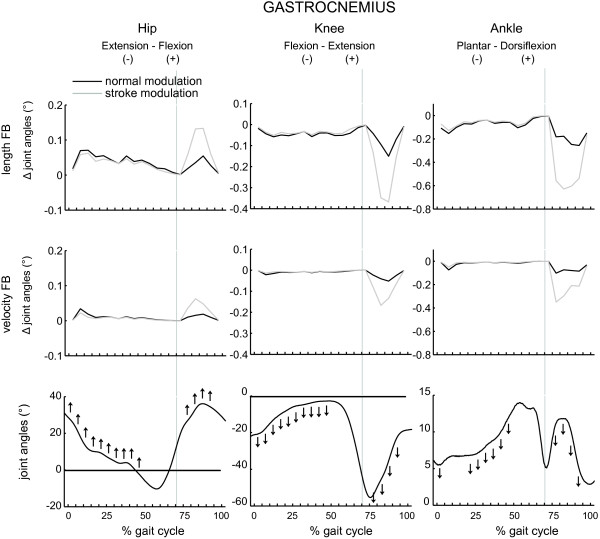
**The effect of increased feedback and altered modulation patterns of gastrocnemius on joint kinematics as function of the gait cycle.** Please find the description in the caption of Figure 5.

**Figure 7:**
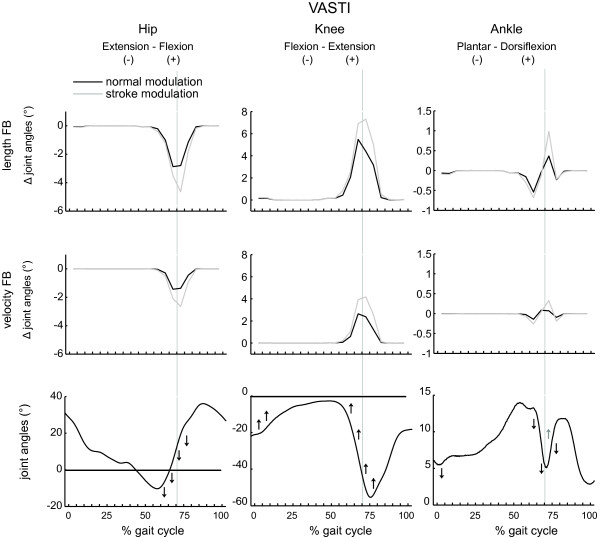
**The effect of increased feedback and altered modulation patterns of vasti on joint kinematics as function of the gait cycle.** Please find the description in the caption of Figure 5.

**Figure 8:**
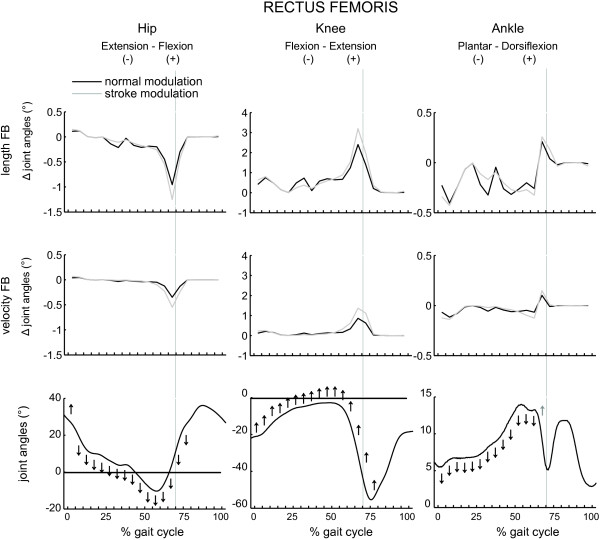
**The effect of increased feedback and altered modulation patterns of rectus femoris on joint kinematics as function of the gait cycle.** Please find the description in the caption of Figure 5.

The results are shown in Figures 5, 6, 7, 8 and Table 3. The figures show the quantitative mean differences in joint kinematics between the reference and stroke kinematics for increased length feedback (top panes) and velocity feedback (middle panes), with normal or with altered modulation patterns. The bottom panes show the reference kinematics (calculated with CMC), and directional changes with increased length/velocity feedback are qualitatively indicated by arrows. Mean differences between reference and simulated stroke muscle excitations can be found in Additional file 4 (SOL + GAS) and Additional file 5 (VAS + RF).

**Table 3:** Schematic overview of the qualitative effects on joint kinematics

	**Hip flexion**				**Knee flexion**				**Ankle dorsiflexion**			
	**SOL**	**GAS**	**VAS**	**RF**	**SOL**	**GAS**	**VAS**	**RF**	**SOL**	**GAS**	**VAS**	**RF**
**Initial contact**		**↑**	**↓**	**↑**		**↑**	**↓**	**↓↓**		**↓**	**↓**	**↓↓**
**Loading response**	**↓**	**↑**	**↓**	**↓**	**↓**	**↑**	**↓**	**↓**	**↓**			**↓**
**Mid stance**	**↓**	**↑**		**↓**	**↓**	**↑**		**↓**	**↓**	**↓**		**↓**
**Terminal stance**	**↓**	**↑**		**↓**	**↓**	**↑**		**↓↓**	**↓**	**↓**		**↓**
**Pre swing**	**↓**		**↓↓**	**↓↓**	**↓**		**↓↓**	**↓↓**	**↓**		**↓↑**	**↓**
**Initial swing**		**↑↑**	**↓↓**	**↓↓**		**↑↑**	**↓↓**	**↓**	**↓↓**	**↓↓**		
**Mid swing**	**↓**	**↑↑**			**↓↓**	**↑↑**			**↓↓**	**↓↓**		
**Terminal swing**										**↓**		

↑indicate increased flexion/reduced extension, ↓ indicate reduced flexion/increased extension for increased length and velocity feedback. The grey arrows (**↑ or ↓)** show the additional effects of the altered modulation patterns.

### Soleus

With increased length feedback gain in the soleus muscle and in the presence of normal reflex modulation, ankle dorsiflexion, knee flexion and hip flexion decreased during loading response, mid- and terminal stance and preswing, compared to the reference condition. During initial and mid-swing, ankle dorsiflexion and knee flexion substantially decreased.

Increased velocity feedback decreased knee flexion and hip flexion during loading response, mid stance.

The presence of an altered modulation pattern emphasized the effect of length and velocity feedback on ankle and knee kinematics during initial and mid swing. Additionally, with altered feedback; a decrease in hip flexion was also found during mid swing.

### Gastrocnemius

Increased length feedback gain induced ankle plantarflexion, during swing, prior to initial contact and continuing at initial contact. During mid- and terminal stance, ankle dorsiflexion slightly decreased. In contrast with the effect of increased soleus length feedback gain, an increase in knee and hip flexion was found at initial contact, and during loading response, mid and terminal stance. Additionally, an increase in hip and knee flexion was found during initial and mid swing.

Increased velocity feedback had a similar effect during the swing phase on joint kinematics compared to increased length feedback.

The altered modulation pattern emphasized the effect of increased length and velocity feedback during swing. In combination with altered feedback patterns, maximum effect of increased length feedback was reached during late mid swing, while with increased velocity feedback, peak effect occurred at early mid swing, and decreased already during late mid swing.

### Vasti

With increased length feedback gain, knee flexion slightly decreased during initial contact and loading response. Likewise, knee flexion decreased during pre and initial swing. Hip flexion decreased during the same gait phases. Excessive hip extension was found during preswing. Ankle plantarflexion slightly increased during initial contact. Dorsiflexion also decreased during pre- swing.

The effect of increased velocity feedback on joint kinematics was similar to increased length feedback.

The altered reflex modulation pattern slightly increased the effect of length and velocity feedback on hip and knee kinematics. However for the ankle, increased feedback in the presence of an altered modulation pattern decreased ankle plantarflexion during stance to swing transition.

### Rectus femoris

Increased length feedback gains decreased knee flexion during the majority of the gait cycle (i.e. from initial contact until initial swing). Hip flexion increased just after loading response, but decreased compared to the reference condition from halfway mid stance till halfway terminal stance. Thereafter, excessive hip extension was found and persisted as decreased hip flexion during initial swing. Increased plantarflexion was found at initial contact and during loading response. During the remainder of stance, ankle dorsiflexion deceased.

With increased velocity feedback gains, the effect on kinematics was less pronounced compared to increased length feedback.

Altered modulation patterns slightly amplified the effect on knee and hip kinematics during initial contact and stance to swing transition. The effect on ankle kinematics was enhanced during initial stance, but decreased during the other phases of the gait cycle.

## Discussion

In the literature, it is generally accepted that muscle weakness contributes to post-stroke gait impairments. However, it is often disputed whether increased length or velocity feedback contribute to spasticity and the consequent gait impairments [9,23,25,42].

This study generated forward simulations using a neuro-musculoskeletal model, i.e. a musculoskeletal model extended with muscle spindle feedback, to investigate the specific effect of increased length and/or velocity feedback on joint kinematics. We hypothesized that hyperexcitability of the muscle spindle reflex loops can induce the specific gait characteristics often found in hemiparetic gait. Furthermore, presence of altered reflex modulation was hypothesized to further emphasize these features.

Our results show that both increased length and velocity feedback of the targeted muscles can induce specific gait impairments often reported in hemiparetic gait and previously associated with hyperexcitability of the reflex loop.

With increased soleus feedback, ankle dorsiflexion decreased during mid-and terminal stance which is in agreement with previous observations of an inadequate dorsiflexion in hemiplegic patients ([18], type I group in [1], extended group in [5], [43]). It corresponds with a decreased forward tibia rotation around the ankle (ankle rocker), which was suggested to thrust the knee into hyperextension, and secondary limit forward progression [1,18,44]. In our results the decreased dorsiflexion caused by increased feedback in soleus was indeed accompanied by a decreased flexion of the knee during stance. Interestingly, the typical stroke features during swing, i.e. an inadequate ankle dorsiflexion and knee flexion, were emphasized even more by introduction of the altered modulation pattern. These impairments in kinematics are often observed in hemiplegic gait [5,43] and also contribute to a hampered toe clearance during swing. In the literature, this observation is mainly related to pretibial muscle weakness and the potential role of hyperreflexia is not specifically considered [18,44]. However, den Otter et al. [45] found a longer activation of TA during the swing phase, which might be necessary to overcome an increased resistance, either originating from mechanical [46] or from neurological factors as hyperexcitability of muscle spindle reflex loops (as indicated by our results). Contribution to inadequate knee flexion is in agreement with results from Little et al. [47], who indicated that a dysfunction of hip-knee coupling is responsible for the impaired paretic toe clearance, rather than an impaired dorsiflexion.

With increased gastrocnemius length and velocity feedback, a similar effect as with increased soleus feedback was found at the ankle level. Moreover, with increased gastrocnemius feedback excessive plantarflexion was found just before initial contact which continued during initial contact itself. This is in accordance with the foot-flat or toe first landing often seen in hemiplegic gait [1,42,43]. In contrast with the effect of the uni-articular soleus muscle, the effect of increased gastrocnemius feedback resulted in an increased hip and knee flexion during the stance phase and during swing. This type of gait impairments corresponds with the ‘flexed’ group as described by Mulroy et al. [5]. In the literature, an increased hip and knee flexion have been attributed to several factors including hamstrings spasticity, quadriceps weakness and PF weakness [18,44]. However, our analysis now shows that gastrocnemius spasticity might also contribute to these gait deficits.

Increased feedback from vasti resulted in a decreased hip and knee flexion and an increased PF during loading response, therefore compromising shock absorption. This is in agreement with the impaired hemiparetic kinematics at foot strike described by Burdett et al. [43]. Furthermore, the increased feedback inhibits an adequate pre- and initial swing knee and hip flexion, necessary for limb advancement. The observed decrease in knee flexion also supports the hypothesis that vastus spasticity contributes to stiff knee gait, as suggested by Goldberg et al. [17]. The altered reflex modulation pattern even reinforces these effects. Additionally, in the presence of altered modulation, the effect of increased feedback on ankle kinematics also reversed: now a decreased plantarflexion is observed during stance to swing transition, consistent with the decreased plantarflexion at toe off found by Burdett et al. [43].

Increased feedback of rectus femoris had a similar effect on the kinematics as described for vasti. However, its effect persists during the entire stance phase, whereas the effect of vasti feedback is limited to initial contact and loading response. In contrast to the role of rectus femoris in inducing hip flexion, the increased feedback only induces an increased hip flexion just after initial contact. Surprisingly, it then induces increased hip extension during the remainder of stance phase. This is conform with the results of a previous simulation study indicating RF to be an indirect contributor to hip extension [6]. Similar to vasti, spasticity of rectus femoris contributes to stiff knee gait, as suggested by the decreased knee flexion during pre, initial and mid swing with increased feedback. Indeed, reduction of rectus femoris spasticity by botulinum toxin injections improves knee flexion during swing [4,48]. It is also in agreement with results from Reinbolt et al. [49], who also showed that pre-swing rectus femoris activity contributes to stiff-knee gait.

As muscle length and muscle lengthening velocity are the prime determinants of the feedback signal originating from the neural control model, we expected a difference in timing of both types of feedback due to timing differences in the maximal muscle length or lengthening velocity in the gait cycle. These timing differences could then potentially discriminate the dominance of either length or velocity feedback in certain phases of the gait cycle. Surprisingly, our results showed only subtle temporal differences between length and velocity feedback; i.e. the most prominent effects on kinematics occur in similar time intervals and only the time evolution between different bins is slightly different for length and velocity feedback. Based on this observation, we cannot attribute the effects seen in kinematics uniquely to a specific type of feedback.

Interestingly, our simulation results suggest that the central reflex modulation has a key role in influencing the kinematic changes induced by the reflex activity: the influence of both length and velocity feedback was either suppressed or enhanced therefore affecting the specific effects of that muscle on the gait kinematics. This lends support to the approach taken by some authors to investigate reflex modulation during gait to assess improvement in gait following gait training for spastic patients [50].

### Limitations

#### *Neural control model*

In the literature, a large ambiguity exists on the contribution of length and velocity feedback to the total muscle excitations even in a normal gait pattern. Contributions of soleus velocity feedback range from no [51] or weak contribution [39], to percentages of 30% to 60% [52]. Based on an unloading experiment, Sinkjaer et al. [51] suggested a major contribution (50%) of group II-length and/or Ib afferents to soleus activity during gait. However, a later study [53] suggested that not length feedback, but only force feedback contributes to muscle activity in human gait. Mazarro et al. showed that both group Ia [54] and group II [55] afferents contribute to soleus activity during normal gait. However no conclusions were made with regard to the absolute contribution of both. In patients with spastic stroke, hyperexcitability of both the monosynaptic stretch reflex and polysynaptic MLR [23,25] was described, implying an important role both for Ia and for group II afferents. As no consensus exists, we made specific assumptions with respect to certain parameters in our neural model. Firstly, length and velocity feedback gains for the reference condition were arbitrarily chosen to generate a feedback without changing the total muscle excitations of the reference simulations. Secondly, to simulate increased feedback, reference gain factors were then multiplied with respectively 1.1, 1.2, 1.5, 3 and 6. As both types of gain factors are chosen arbitrarily, we cannot make statements on the absolute effect of the increased feedback on joint kinematics, nor on the relative contribution of length versus velocity feedback.

The modulation curves we implemented are based on literature. These are only reported in a very limited number of patients [26,27]. Therefore, we cannot exclude that, just as there are different types of gait patterns in stroke patients [1,5], different modulation patterns exist in subgroups of patients. Furthermore, modulation curves of quadriceps are based on tendon tap reflexes, while the curves for PF are based on H-reflex measurements, which might have been a confounding factor. However, it was shown in previous studies that both H-reflexes [13] and tendon-tap reflexes [14] in quadriceps show a similar modulation pattern during gait in healthy subjects. According to Faist and Berger (unpublished data), SOL H-reflex and tendon-tap reflexes are modulated also similarly in hemiplegic subjects. Therefore no large differences are expected between H-reflex and tendon tap quadriceps reflex modulation in hemiplegic subjects.

The focus of our study was mainly the effect of increased muscle spindle feedback on gait impairments. It is recognized that other reflex pathways, e.g. Ib force feedback pathways or cutaneous feedback are also very important in the regulation of gait [56], and might therefore also be involved in the impaired gait pattern often seen in hemiplegic patients. However the study of these contributions is left for further investigation.

Base excitations and initial states are determined from a simulation of normal gait, which allows us to evaluate the unique effect of increasing feedback and altered reflex modulation patterns. This way, the effect of altered feedback is isolated from other concomitant neural changes, like decreased central neural drive, which might also change motor neuron activity. Using the same base excitations also implies that we do not take into account the interaction effect of a changed background activity on the reflex modulation patterns. It is generally known that modulation of reflexes during the gait cycle depends both on background level and on a central modulation factor. Faist et al. [26] assessed the influence of background EMG by comparing quadriceps reflexes during gait with reflexes elicited during standing with a similar EMG activity and knee angle. Independent of background EMG, different modulation curves were found in hemiplegic patients compared to healthy subjects. This suggests that the altered modulation pattern is primarily due to altered central modulation during walking. In our opinion, it allowed us to use the altered modulation patterns in combination with the same base excitations to predict the effect on kinematics. However, by using the same base excitations our model does not allow predicting compensatory strategies adopted by hemiparetic subjects or to evaluate the effect of a decreased central neural drive. Future neuro-musculoskeletal models should therefore concentrate on including both altered central neural drive and altered feedback to investigate their combined effect on gait kinematics.

The neural control model in this study includes length and velocity feedback for muscles with a predominant function in the sagittal plane, consequently the effect in mediolateral direction i.e. hip abd/adduction and exo/endorotation was expected to be small. Therefore, although we generated three-dimensional simulations, we chose to only report the effect on sagittal plane joint kinematics.

### Foot-ground contact model

A sensitivity analysis of the different parameters in the model showed that the position of the spheres within the local coordinate frame of the foot were most important to improve the kinematic tracking. We therefore opted to optimize these locations in order to minimize the kinematic tracking error.

The other parameters of the elastic foundation contact model were estimated. Our validation results showed already good agreement (Figure 4) between forward simulations using measured GRF and the foot-ground contact. However, in future research, we consider optimization of stiffness, dissipation and friction parameters to further enhance the validity of the foot-ground contact model.

## Conclusions

The effects of increased length and velocity feedback in combination with altered reflex modulation patterns on joint kinematics are in accordance with previously reported gait deviations in hemiparetic patients. Hence, in agreement with our hypothesis, the results support the idea that hyperexcitability of length and velocity feedback pathways underlie some of these reported gait deviations. Furthermore the data show indeed that the altered modulation patterns play an important role, especially during the swing phase. In contrast, no conclusion could be drawn concerning the relative contribution of length or velocity feedback. For the latter, more experimental research is necessary in the future to provide reflex modulation patterns of a more extended group of stroke patients. Secondly there is a need for more experimental data on the relative gains of length and velocity feedback loops in these patients.

## Competing interests

The authors declare that they have no competing interests.

## Authors’ contributions

KJ performed the experimental data collection, contributed to the model implementation, executed simulations, analyzed data and interpreted the data. FDG implemented the simulation model, contributed to data analysis and interpretation. IJ contributed to the model implementation, data analysis and interpretation. WA was involved in the implementation of the foot-ground contact model. JDS, JD and IJ are senior authors who participated in the conception and design of research and helped to draft the manuscript. All authors read and approved the final manuscript.

## Supplementary Material

Additional file 1 Ground reaction forces of the 1 km/h reference simulation in anterior-posterior, vertical and up-down direction are shown as function of the gait cycle. Click here for file

Additional file 2 Kinematics (degrees) of the 1 km/h reference simulation are shown for trunk and pelvis, and bilaterally for hip, knee and ankle joints (left: grey, right: black) as function of the gait cycle. Click here for file

Additional file 3 **Comparison of experimentally measured electromyography signals (EMG) and simulated muscle activations (from CMC) during the 1 km/h reference simulation.** Both EMG and simulated activations are normalized to the maximum value over the gait trial. EMG/activations are shown for biceps femoris (BF), semitendinosus (ST), rectus femoris (RF), lateral vastus (LVAS), lateral gastrocnemius(LGAS), soleus (SOL), and tibialis anterior (TA). Click here for file

Additional file 4 **The effect of increased feedback (FB) and altered modulation patterns of A. Soleus, B. Gastrocnemius on muscle excitation as function of the gait cycle.** The mean differences are shown between the reference and stroke excitation for increased length FB and velocity FB, with normal modulation patterns (‘normal’, black line) or with altered modulation patterns (‘stroke’, grey line). The bottom panes show the reference excitations (calculated with CMC). The grey vertical line indicates stance-to-swing transition. Click here for file

Additional file 5 **The effect of increased feedback (FB) and altered modulation patterns of A. Rectus femoris, B. Vasti on muscle excitation as function of the gait cycle.** The mean differences are shown between the reference and stroke excitation for increased length FB and velocity FB, with normal modulation patterns (‘normal’, black line) or with altered modulation patterns (‘stroke’, grey line). The bottom panes show the reference excitations (calculated with CMC). The grey vertical line indicates stance-to-swing transition. Click here for file
