# Supplementary figures and images for: Altering length and velocity feedback during a neuro-musculoskeletal simulation of normal gait contributes to hemiparetic gait characteristics
Source: J Neuroeng Rehabil. 2014 Apr 30;11:78. doi: 10.1186/1743-0003-11-78 (PMC4030738; doi:10.1186/1743-0003-11-78)

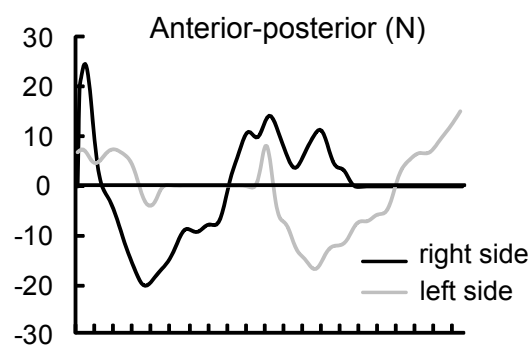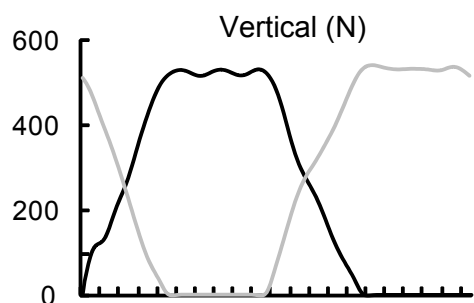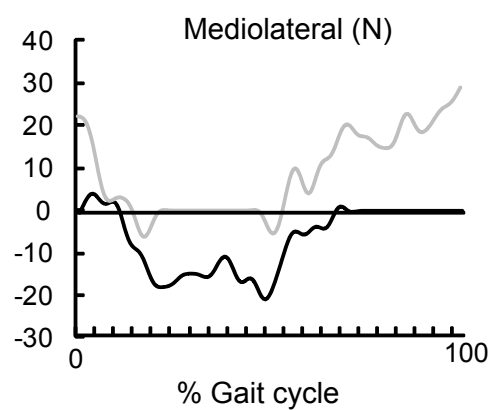

Supplement: Additional file 1 — Ground reaction forces of the 1 km/h reference simulation in anterior-posterior, vertical and up-down direction are shown as function of the gait cycle. [file 1743-0003-11-78-S1.pdf]

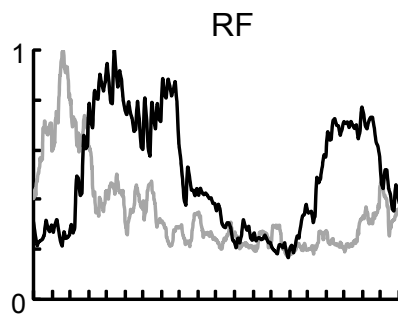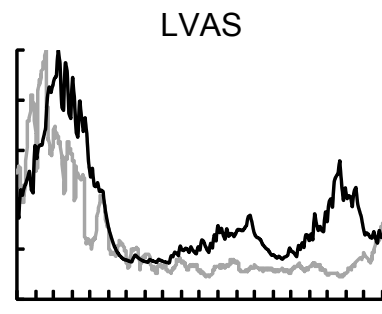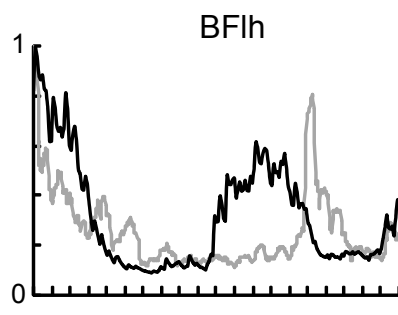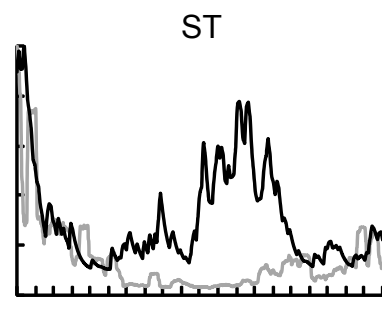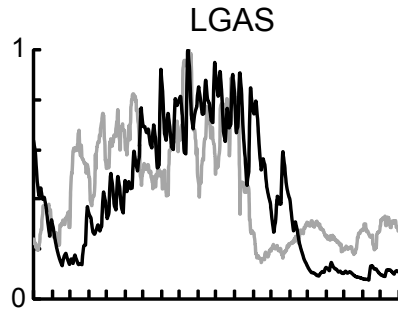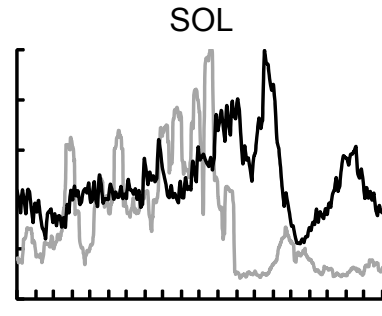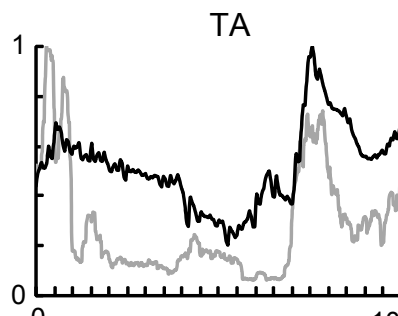

— simulated activation  
— experimental EMG

% Gait cycle

Supplement: Additional file 3 — Comparison of experimentally measured electromyography signals (EMG) and simulated muscle activations (from CMC) during the 1 km/h reference simulation. Both EMG and simulated activations are normalized to the maximum value over the gait trial. EMG/activations are shown for biceps femoris (BF), semitendinosus (ST), rectus femoris (RF), lateral vastus (LVAS), lateral gastrocnemius(LGAS), soleus (SOL), and tibialis anterior (TA). [file 1743-0003-11-78-S3.pdf]

## SOLEUS

**A**

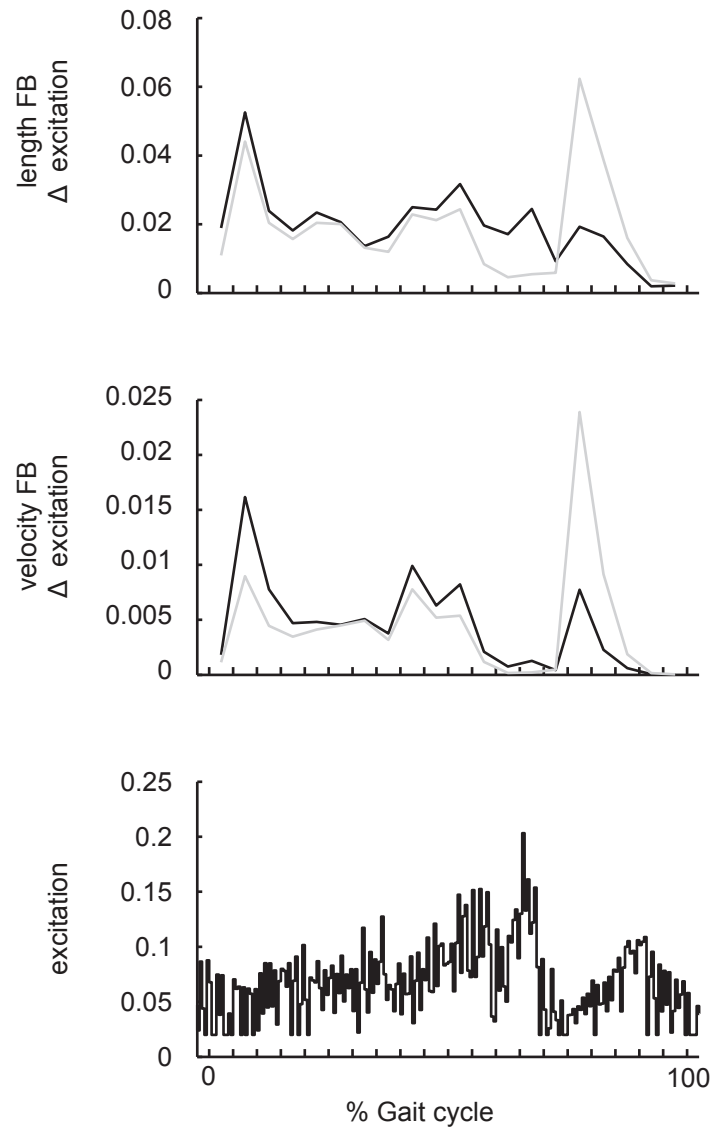

## GASTROCNEMIUS

**B**

medial

lateral

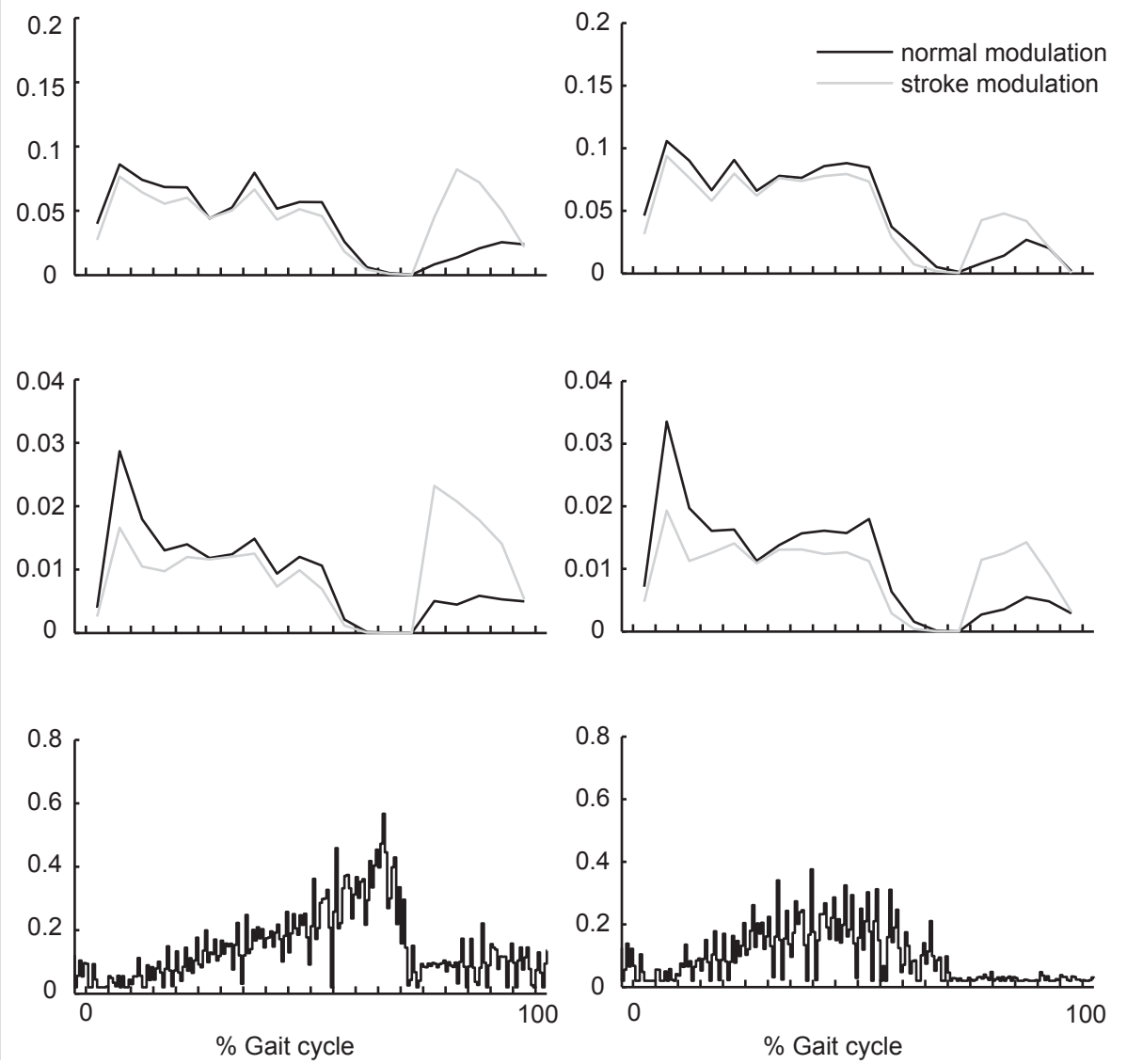

Supplement: Additional file 4 — The effect of increased feedback (FB) and altered modulation patterns of A. Soleus, B. Gastrocnemius on muscle excitation as function of the gait cycle. The mean differences are shown between the reference and stroke excitation for increased length FB and velocity FB, with normal modulation patterns (‘normal’, black line) or with altered modulation patterns (‘stroke’, grey line). The bottom panes show the reference excitations (calculated with CMC). The grey vertical line indicates stance-to-swing transition. [file 1743-0003-11-78-S4.pdf]
