# Supplementary material for: Altering length and velocity feedback during a neuro-musculoskeletal simulation of normal gait contributes to hemiparetic gait characteristics
Source: J Neuroeng Rehabil. 2014 Apr 30;11:78. doi: 10.1186/1743-0003-11-78 (PMC4030738; doi:10.1186/1743-0003-11-78)
Supplement: Additional file 2 — Kinematics (degrees) of the 1 km/h reference simulation are shown for trunk and pelvis, and bilaterally for hip, knee and ankle joints (left: grey, right: black) as function of the gait cycle. [file 1743-0003-11-78-S2.pdf]

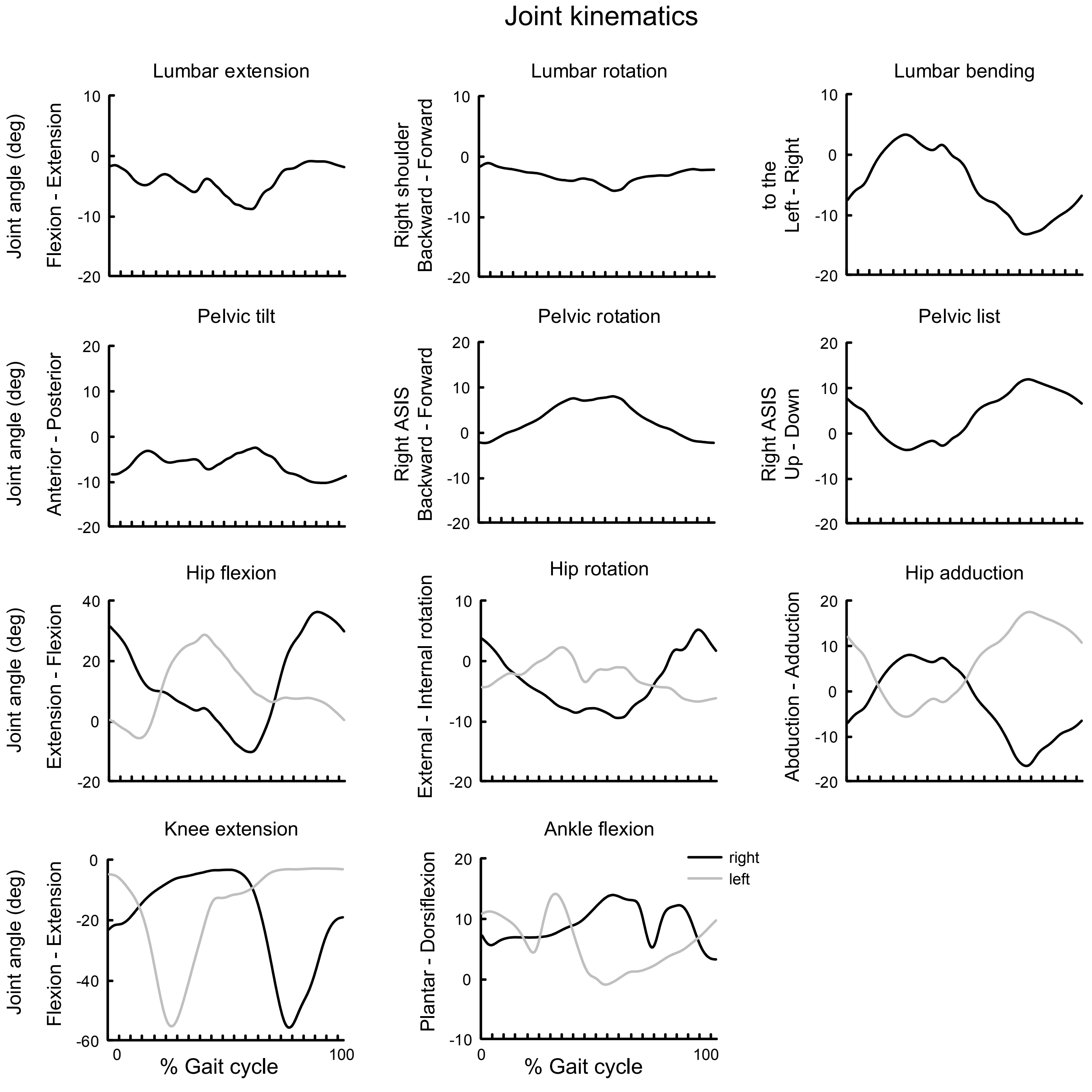
Joint kinematics
Lumbar extension
Lumbar rotation
Lumbar bending
Pelvic tilt
Pelvic rotation
Pelvic list
Hip flexion
Hip rotation
Hip adduction
Knee extension
Ankle flexion
Joint angle (deg)
Flexion - Extension
Right shoulder
Backward - Forward
to the
Left - Right
Anterior - Posterior
Right ASIS
Up - Down
Extension - Flexion
External - Internal rotation
Abduction - Adduction
Plantar - Dorsiflexion
right
left
% Gait cycle
0
100
10
0
-10
-20
20
40
-40
-60
